# Magnetic resonance imaging-guided single-fraction preoperative radiotherapy for early-stage breast cancer (the RICE trial): feasibility study

**DOI:** 10.1186/s40814-024-01557-6

**Published:** 2024-11-07

**Authors:** Ayyaz Qadir, Nabita Singh, Jenna Dean, Kerryn Brown, Mark Tacey, Bruce Mann, Tomas Kron, Glenn Cahoon, Eddie Lau, Andrew M. Scott, Belinda Yeo, Su-Wen Loh, Sergio Uribe, Aung Aung Kywe Moe, Kerryn Ireland-Jenkins, Rosly McAuley, Leah McDermont, Wei Ming Ooi, Suat Ng, Michael Chao, Farshad Foroudi

**Affiliations:** 1 Department of Medical Imaging and Radiation Sciences, School of Primary and Allied Health Care, Monash University, Melbourne, Australia; 2 Department of Radiation Oncology, Newton-John Cancer Wellness and Research Centre, 145 Studley Road, PO Box 5555, Heidelberg , Austin HealthVictoria, Olivia 3084 Australia; 3 Royal Women’s Hospital, Parkville, VIC Australia; 4 Melbourne University, Parkville, VIC Australia; 5 Peter MacCallum Cancer Centre, Parkville, VIC Australia; 6 Austin Health, Heidelberg, VIC Australia; 7 School of Cancer Medicine, Olivia Newton John Cancer Research Instituteand, Latrobe University , Melbourne, VIC Australia; 8 Department of Molecular Imaging, Austin Health and University of Melbourne, Victoria, Australia; 9 Medical Oncology, Austin Health, Heidelberg, VIC Australia; 10 Breast Surgery, Austin Health, Heidelberg, VIC Australia; 11 Anatomical Pathology, Austin Health, Heidelberg, VIC Australia

**Keywords:** Breast cancer, Magnetic resonance imaging, Radiation therapy, Response, Study protocol, Neoadjuvant therapy

## Abstract

**Background:**

Over the past decade, the adoption of screening programs, digital mammography, and magnetic resonance imaging (MRI) has increased early-stage breast cancer diagnosis rates. Mortality rates have decreased due to early detection and improved treatments, including personalized therapies. Accelerated partial-breast irradiation (APBI) is emerging as a convenient and effective treatment for some patients, with studies exploring its preoperative use. Preoperative APBI, especially with MRI guidance, offers improved tumor targeting and potentially reduced side effects. Magnetic Resonance Imaging-Guided Single-Fraction Pre-Operative Radiotherapy for Early-Stage Breast Cancer (RICE trial) aims to assess the feasibility and efficacy of MRI-guided single-dose radiotherapy (RT) for early-stage breast cancer.

**Methods:**

The RICE study is a prospective, single-arm study evaluating single-fraction preoperative, APBI treatment for patients with early-stage breast cancer using a magnetic resonance imaging linear accelerator (MRI linac). Eligible patients enrolled in this study will have a core biopsy to confirm estrogen receptor-positive and HER2-negative sub-type. RT planning will use a planning computed tomography (CT) co-registered with a MRI with the patient in either the supine or prone position. For the diagnostic workup, [18F] fluorodeoxyglucose positron emission tomography/CT ([18F] FDG PET/CT) and [18F] fluoroestradiol positron emission tomography/CT ([18F] FES PET/CT) will be performed prior to treatment. Thirty patients will receive a single ablative RT dose of 21 Gray to the tumor. Pre-treatment and post-treatment MRI scans will be acquired at baseline and 5 weeks post-RT respectively. Breast-conserving surgery will be scheduled for 6 weeks after APBI treatment using the MRI linac.

The primary study endpoint is the successful administration of a single fraction of preoperative breast RT under the guidance of an MRI linac. Secondary endpoints include evaluating the utility of MRI, [18F] FDG PET/CT, and [18F] FES PET/CT as a non-invasive method for assessing treatment response in patients undergoing single-fraction preoperative APBI.

**Conclusion:**

The RICE trial represents a significant step in breast cancer treatment, offering insights that could lead to treatment protocols with minimized RT appointments and enhanced patient outcomes.

**Trial registration:**

This trial is registered with the Australian New Zealand Clinical Trials Registry (ANZCTR). Registered 31st of May 2021. Registration number: ACTRN12621000659808.

## Background

Over the past decade, the implementation of screening programs and the adoption of digital mammography and magnetic resonance imaging (MRI) have led to an increased incidence of early-stage breast cancer diagnosis. This trend is expected to continue, driven by the increasing prevalence of risk factors, population growth, and aging [1, 2]. Despite this, mortality from early-stage breast cancer has significantly decreased, with the global 5-year net survival rate estimated to increase from 67.9 to 78.2% [3, 4]. Simultaneously, there is a shift towards personalized and less invasive therapies, aimed at de-escalating therapy, improving cosmetic outcomes, and reducing radiotherapy (RT)-related toxicity, all while ensuring the safety and efficacy of oncological care [4–8].

Currently, patients diagnosed with early-stage breast cancer typically undergo wide local excision, followed by daily postoperative whole-breast irradiation lasting anywhere from 3 to 5 weeks [9–13]. While this regimen effectively reduces tumor recurrence and decreases breast cancer mortality, the conventional post-operative treatment schedule of 3–5 weeks is prohibitively long for many patients, particularly among the elderly, rural residents, and those living in underserved regions [14, 15]. In an effort to provide greater convenience and improve the quality of life for patients and also considering that up to 90% of recurrences are found close to the lumpectomy site, hypo fractionated accelerated partial-breast irradiation (APBI) is currently being explored. APBI, a technique in which RT is delivered solely to the high-risk tissue, has emerged as a promising alternative to whole-breast irradiation [11, 16, 17]. Several phase III trials using different APBI techniques have demonstrated that, for selected patients, APBI achieves satisfactory local control comparable to that of whole-breast irradiation, but with fewer side effects, greater convenience, better quality of life, and reduced costs [7, 18, 19, 20, 21, 22, 23]. As such, APBI has recently become a standard treatment for low-risk patients [4, 7, 19, 20]. In general, patients are eligible when they meet the suitability criteria established by The European Society for Radiotherapy (ESTRO) or the American Society for Radiation Oncology (ASTRO) [4, 18, 22].

Ensuring the accurate delivery of APBI is critical, as it targets only the high-risk tumor tissue and not the entire breast. However, postoperative APBI presents challenges due to difficulties in precisely delineating the tumor from the surrounding healthy tissue, which can be affected by breast distortion and the presence of postoperative seroma [4]. Consequently, this often leads to less precise target volume definition and greater variability in delineation among Radiation Oncologists, resulting in unnecessarily large irradiated volumes [20, 24, 25].

In an effort to reduce the irradiated volume and improve cosmetic outcomes, studies are now investigating the potential use of APBI in the preoperative setting [5, 23, 25–30]. One promising alternative is preoperative magnetic resonance-guided radiotherapy (MRgRT). With the exceptional soft tissue contrast provided by MRI, delivering preoperative APBI using a magnetic resonance-integrated linear accelerator (MRI linac) allows for greater distinction between the tumor and surrounding healthy tissue, as the tumor remains in its original location [4, 6, 28, 31]. This approach leads to reduced inter-observer variability in contouring the tumor and thus enhanced definition of the tumor volume [4, 6, 28]. Furthermore, since a smaller volume of tissue is treated, preoperative APBI enables the delivery of a higher dose per fraction. This facilitates the possibility of single-dose RT treatment, further reducing the treatment burden and RT-induced toxicity to the patient, thereby significantly improving the patient's quality of life [4–8].

Another advantage of preoperative RT is the ability to noninvasively assess treatment response via imaging. Currently, in the setting of preoperative systemic therapy, treatment response assessment is performed using MRI [4, 32, 33]. However, data on treatment response assessment with MRI after preoperative RT are limited. Notably, two separate studies reported that MRI has a positive predictive value (PPV) ranging from 67 to 88% and a negative predictive value (NPV) ranging from 76 to 85% in predicting pathological complete response [4, 27, 28]. Although these studies were significantly underpowered, and more data are needed to establish the value of MRI in developing a prediction model for pathologic response, if a pathologic complete response can be more accurately predicted after preoperative APBI in the future, surgery could potentially be omitted in these low-risk patients. For patients without a pathologic complete response, single-fraction RT could replace post-operative RT schedules, and tumor downstaging leading to less healthy breast tissue being excised and improve the overall cosmetic outcome.

In the single-institution prospective study in Melbourne, Australia, which we call the Magnetic Resonance Imaging-Guided Single-Fraction Pre-Operative Radiotherapy for Early-Stage Breast Cancer (RICE) trial, our goal is to assess the feasibility of MRI-guided single-dose preoperative APBI using an MRI linac as a definitive treatment for early-stage breast cancer. We also aim to investigate the utility of MRI as a non-invasive tool for assessing treatment response after single-fraction neoadjuvant RT.

## Methods/design

### Study design

This single-arm prospective trial will be conducted at the Radiation Oncology Department of Austin Health, Melbourne, Australia.

### Primary and secondary endpoints

The primary endpoint of the study is to assess the feasibility of administrating a single fraction of preoperative RT under the guidance of an MRI linac. Feasibility is defined as achieving at least 90% participation among eligible and consenting participants who receive the full prescribed radiation dose under MRI linac guidance.

The secondary objective is to evaluate the relationship between pre-RT MRI and post-RT MRI with respect to histopathological findings.

### Patient recruitment and selection

Prospective participants for the study will be identified through two main channels: multidisciplinary meetings and direct referrals from external specialists to the Radiation Oncology Department.

Table 1 presents an overview of the inclusion and exclusion criteria, which align with the guidelines for APBI set forth by ASTRO and ESTRO [18, 34].

**Table 1:** Overview of the inclusion and exclusion criteria for the RICE trial

Inclusion	Exclusion
Age ≥ 60 years	Previous radiotherapy to the same breast
Unifocal	Suspicious diffuse microcalcifications
Tumor visible on pre-treatment imaging	Invasive lobular carcinoma on core biopsy
Tumor size on pre-treatment imaging ≤ 2 cm	Lymphovascular invasion on core biopsy
Grade 1–2 on pre-treatment core biopsy	Any clinical nodal or metastatic disease
Estrogen receptor (ER) and progesterone (PR) positive (> 10%)	Human epidermal growth factor receptor 2 (HER2) positive
Clinically node-negative	Prior non-hormonal therapy
Breast primary tumor visible on MR radiation oncology imaging (with or without IV contrast) for treatment localization	Skin or chest wall involvement
	Collagen vascular disease (e.g., lupus, scleroderma)
	Pregnant or lactating
	Absolute contraindications to MRI (e.g., incompatible implants, unable to tolerate duration)

### Informed consent

If a subject is considered eligible based on this preliminary screening, the surgeon will provide detailed information about the clinical study. Patients interested will receive additional information from the Radiation Oncology trial coordinator. Furthermore, patients will be referred to the Radiation Oncologist at the Olivia Newton-John Cancer Center for preoperative consultation and receive further information about the RT treatment.

### Procedure

**Fig. 1:**
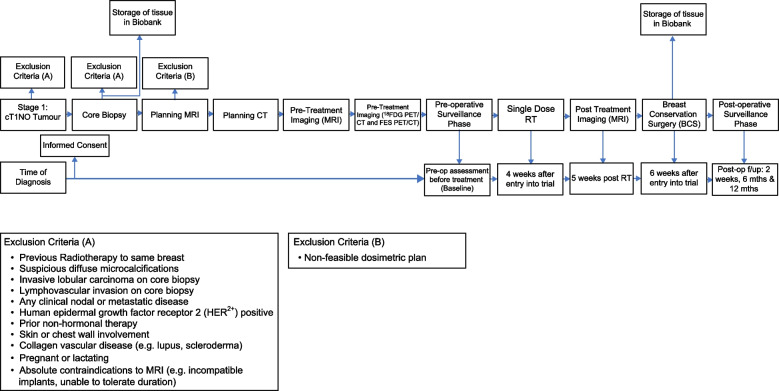
Overview of the study procedures for the trial

An overview of the study procedures for the trial is illustrated in Fig. 1.

Upon entering the trial, patients will first undergo a clinical examination and core biopsy. This will be followed by treatment planning, which will involve MRI and CT simulation. For the purpose of RT planning and diagnostic workup, [18F] FDG PET/CT and [18F] FES PET/CT images of both breasts will be acquired.

Six weeks after receiving RT treatment, patients will undergo wide local excision along with sentinel lymph node biopsy.

### Pre-treatment surveillance imaging

The baseline assessment of treatment response will be conducted on an MRI simulator system (Phillips Ingenia Ambition 1.5 T MRI) utilizing the department’s 1.5 T system. The MRI is equipped with a dStream 7 Channel dedicated breast coil from Phillips Healthcare (Best, Netherlands). For this assessment, patients will assume a prone position.

### MRI linac and RT for breast sites

The Elekta Unity MR-linac (Elekta, Stockholm, Sweden) is a combination of a modified Philips Ingenia 1.5 T MRI with a split-coil superconducting magnet and a linear accelerator. The beam generation system produces a single 7-megavoltage flattening filter-free X-ray source and is mounted on an annular gantry that freely rotates around a cylindrical cryostat containing the static-field MR coils. The Unity radiation field is shaped by a multi-leaf collimator, ranging from 0.8 cm × 0.5 cm to 57.4 cm × 22.0 cm at the isocenter. The patient positioning system is capable of only longitudinal movement, making it impossible to move the treatment couch laterally or vertically. The isocenter is 14.0 cm above the patient positioning system and 143.5 cm from the source.

The challenges particularly associated with breast treatment on the MR-linac include geometric accuracy due to gradient non-linearities combined with lateral target volumes, the electron return effect, and the electron stream effect. These issues have been described and addressed by earlier studies [35], and procedures are included in the protocol for this study to minimize their impact on treatment for RICE patients. These procedures include the use of distortion correction software for geometric accuracy, carefully chosen beam angles, and organs at risk margins to minimize the effects of electron stream effect, as well as the use of bolus material to shield any predicted electron stream effect from treatment planning simulation. Strategies for online plan adaptation compensate for the fixed couch position, and online motion monitoring allows for gating (beam pause) when the target moves out of the planned high-dose volume.

### RT planning

In this study, RT planning will involve both MRI and CT simulation, conducted with the patient in either the supine or prone position. MRI simulation will occur prior to CT to ensure that the lesion is visible on the imaging sequences that are available on the integrated MRI linac, to support treatment verification and delivery. It will also be used to determine whether the patient would benefit from treatment in the supine or prone position without additional exposure to ionizing radiation. The gross tumor volume (GTV) will be contoured as the visible tumor on the CT and MRI scans. The clinical target volume (CTV) will be defined by uniformly expanding the GTV by 15 mm. This expansion will be limited posteriorly by the pectoralis muscles and an anterolateral margin of 5 mm from the skin. The volume will not extend across the midline. The PTV will be created from a 3-mm margin expansion of the CTV, to account for minor inter and intra-fraction variations in patient positioning.

Treatment planning will be completed using Monaco Treatment Planning System (Elekta Stockholm, Sweden) using step-and-shoot Intensity Modulated Radiation Therapy, with multiple beams positioned at angles optimized for target coverage and organ at risk sparing within the current MRI linac capabilities.

For the dose prescription, the plan is to ensure that 95% of the CTV receives the full prescribed single dose of 21 Gy. Additionally, 95% of the PTV is intended to receive at least 95% of the prescribed dose, while a maximum of 2% of the PTV is intended to be exposed to 107% of the prescribed dose. The dose criteria for this study were determined based on conservative consideration of available literature for similar dose prescriptions for this clinical indication [6, 29, 30].

### RT delivery

Following planning, a single 21 Gray (Gy) fraction will be delivered within 10 days of the simulation. Treatment will be administered via an MRI linac (Elekta Unity Sweden). The patient will be positioned according to the RT planning position, and a verification image will be acquired. The image will be assessed, anatomical contours adjusted, and the treatment plan adapted to match the patient’s anatomy. Treatment will be delivered with real-time cine MRI monitoring to ensure safe and accurate delivery of the dose to the PTV.

### Post-treatment surveillance imaging

Post-treatment assessment of the response will be conducted 5 weeks after treatment using the MRI simulator. The same patient positioning and protocol as established in the pre-treatment surveillance imaging step will be used; this consistency will ensure comparability between the pre-treatment and post-treatment images, allowing for accurate evaluation of the effectiveness of treatment.

### Breast-conserving surgery

Six weeks after completing RT treatment, the patients will undergo breast conservation surgery, which consists of wide local excision for removing the tumor, along with sentinel lymph node biopsy to assess for the potential spread of cancer to the lymph nodes. In cases where the sentinel lymph node is found to be positive, indicating potential cancer spread, patients will be counseled regarding the possibility of axillary clearance. The decision for this additional procedure will be made based on the discretion of the treating surgeon, considering the individual patient's condition and treatment needs.

Histopathological assessment of the excised tissue will be performed by a pathologist with a sub-specialization in breast cancer. Tumor cells will be evaluated using hematoxylin and eosin staining and cytokeratin antibody activity. The response to treatment will be graded using the Miller and Payne Reporting Criteria [36].

Tumor samples will be securely stored within the Pathology Health biobank, and blood samples will be stored in the pathology freezer.

### Follow-up

Several clinical assessments, cosmetic photographs, and formal acute toxicity scoring will be conducted by the treating Radiation Oncologist at key time points throughout the study. A baseline assessment will be performed prior to the commencement of treatment; then, follow-up assessments will be performed at 2 weeks, 6 months, and 12 months post-treatment.

The acute toxicity and cosmetic outcome scores will adhere to the standards set by the Common Terminology Criteria for Adverse Events (CTCAE) Version 5.0 [37].

All adverse events resulting from RT experienced by the participants will be documented in the case report form. Importantly, adverse events that could be influenced by the study interventions will be considered outcomes of the study.

### Patient-reported outcomes

Patients will be asked to complete quality of life questionnaires (QLQ-C30 and QLQ-BR23) [38] at four key time points: upon study enrollment, on the day of RT treatment, 2 weeks postsurgery, and at the 12-month follow-up. Additionally, patient-based cosmetic evaluations will also be reported on the questionnaires.

### Sample size

Based on the eligibility criteria, 30 patients are expected to provide consent and be recruited over the 2-year study period. This number of patients will provide an estimated 95% confidence limit of feasibility consisting of a lower bound of 74.4% and an upper bound of 96.5% (based on the Wilson score interval approximation), if a feasibility proportion of 90% is achieved.

### Statistical methods

Descriptive summary statistics will be determined to summarize the patient clinical characteristics, responses to patient-reported outcome questionnaires, and rates of adverse events at each follow-up time point. The primary outcome will be reported as the proportion of patients who can be feasibly treated with MRI linac using a single-fraction APBI technique. The denominator includes all patients who met the study eligibility criteria and provided consent to participate. Success for this outcome will be defined as achieving this treatment outcome in at least 90% of patients. For the secondary outcome assessment, univariate analysis will be performed to investigate the association between MRI parameters and the tumor response rate. Categorical variables (i.e., Clavien–Dindo grade) will be presented as counts and percentage frequencies, while continuous variables (i.e., quality of life or patient-reported outcome measures) will be presented as the mean ± standard deviation if they are normally distributed or the median (interquartile range) if their distribution is skewed.

Pearson or Spearman’s rank correlation coefficients will be used to assess correlations for normally and non-normally distributed continuous variables, respectively. Appropriate parametric or nonparametric tests will be applied to assess associations between the MRI data and histopathological features of the patients. Additionally, the MRI results will be compared between good responders to treatment and poor responders to treatment. Receiver operating characteristic (ROC) curve analysis may be employed to determine cut-off points for evaluating associations between continuous variables and binary outcomes, such as the tumor response rate.

All the statistical analyses will be conducted using Stata version 18.0 (StataCorp, College Station, Texas, USA). A *p*-value of less than 0.05 will be used to indicate statistical significance. However, we recognize that the study may be underpowered to detect statistical significance for some tests of association when assessing the secondary outcomes due to the limited sample size. Given the exploratory nature of the analysis for the secondary outcomes, it is challenging to determine the number of statistical tests that will be conducted a priori. Therefore, we will focus on the magnitude of effect sizes and confidence intervals when reporting and describing the results for the secondary outcomes. This approach is likely to inform sample size calculations for subsequent fully powered trials.

## Discussion

In the RICE trial, we will administer a single fraction of preoperative APBI using the MRgRT on an MRI linac, with the hypothesis that enhanced treatment accuracy and acceptable post-surgical breast cosmesis can be achieved. The MRI linac system’s ability to integrate real-time MRI with a RT treatment machine offers superior tumor-targeting precision over traditional CT-Linac external beam methods. This precise targeting allows accurate tumor radiation while protecting surrounding healthy tissue. Additionally, the single-fraction approach is expected to enhance patient experience and convenience, potentially reducing hospital visits and streamlining treatment.

The optimal dose and timing for removing an irradiated tumor remain debated, with various prospective trials exploring a variety of options, including treatment technique and dose. In our study, we will administer a single 21-Gy fraction, with pathological response assessment planned for 6 weeks after treatment. Given the exploratory nature of this study, our decision to use this dose was based on prior studies [5, 28–30] that have demonstrated its effectiveness without significant RT-related side effects and improved overall quality of life. Given the variability among studies investigating single-fraction APBI [4–6, 28–30], particularly in terms of the time intervals and techniques used, it is anticipated that a portion of patients will demonstrate a response to treatment. Nevertheless, since no study to date has explored, the response to treatment 6 weeks after single-fraction preoperative RT using the MRI linac, these outcomes remain largely unexplored.

Currently, RT schedules are typically uniform across patients’ treatments, despite the variability in treatment response among individuals. Some patients exhibit significant tumor residue post-treatment, while others achieve a near-complete pathologic response. If imaging can accurately differentiate between responders and non-responders before or after RT, it may be possible to tailor treatment more precisely. For potential responders, dose de-escalation could reduce treatment toxicity while maintaining favorable oncological outcomes. Conversely, poor responders might benefit from a more aggressive treatment plan.

In our study, we intend to collect MRI data both before and after treatment to correlate these parameters with radiation response biomarkers. This method aims to explore the potential of MRI as a non-invasive tool for assessing treatment efficacy. Additionally, our study includes baseline [18F] FDG-PET-CT and [18F] FES-PET-CT to determine whether pre-treatment molecular imaging can identify potential RT responders before intervention. Given our inclusion criteria for the RICE trial — ER + sub-type tumors smaller than 2 cm — we have specifically chosen to evaluate the use of [18F] FES-PET-CT. This decision aims to determine whether this radiotracer offers greater sensitivity and specificity compared to the standard [18F] FDG-PET-CT, particularly given the limitations of [18F] FDG-PET-CT in detecting small tumors and its variable sensitivity in ER + sub-types which have low glucose uptake [39–41]. We anticipate that [18F] FES-PET-CT, an ER-sensitive radiotracer, will improve the detection of such lesions and enhance the diagnostic workup for RT planning and treatment.

## Conclusions

In conclusion, the RICE trial is a single-institution prospective study that will contribute to the growing literature on the use of single-fraction preoperative RT in early-stage breast cancer patients. This trial primarily focuses on the feasibility of MRgRT as a novel treatment tool that could potentially represent a significant advancement in early-stage breast cancer treatment and clinical research. The insights and experiences gained from this trial are expected to inform and enhance future research in this area, ultimately leading to improved patient outcomes and care.

## Data Availability

Data and materials will not be made available until the end of the study and upon request.

## Abbreviations

MRgRT: Magnetic resonance imaging-guided single-fraction pre-operative radiotherapy
RICE Trial: Magnetic Resonance Imaging-Guided Single-fraction Preoperative Radiotherapy for Early-Stage Breast Cancer
MRI: Magnetic resonance imaging
WLE: Wide local excision
APBI: Accelerated partial-breast irradiation
MRI linac: Magnetic resonance imaging linear accelerator
18F-FDG PET/CT: [18F] fluorodeoxyglucose positron emission tomography
ER +: Estrogen receptor-positive
PR +: Progesterone receptor-positive
HER2 +: Human epidermal growth factor receptor 2 positive
PTV: Planning target volume
CTV: Clinical target volume
GTV: Gross target volume
CT: Computed tomography
DCE-MRI: Dynamic contrast-enhanced magnetic resonance imaging
CTCAE: Common Terminology Criteria for Adverse Events
Gy: Gray
HREC: Human Research Ethics Committee
PPV: Positive predictive value
NPV: Negative predictive value
RT: Radiotherapy
